# A systems biology approach to understand the pathophysiological mechanisms of cardiac pathological hypertrophy associated with rosiglitazone

**DOI:** 10.1186/1755-8794-7-35

**Published:** 2014-06-17

**Authors:** Lars Verschuren, Peter Y Wielinga, Thomas Kelder, Marijana Radonjic, Kanita Salic, Robert Kleemann, Ben van Ommen, Teake Kooistra

**Affiliations:** 1 TNO, Department Microbiology and Systems Biology, P.O. Box 360, 3704 AJ Zeist, The Netherlands; 2 TNO, Department Metabolic Health Research, Leiden, The Netherlands

## Abstract

**Background:**

Cardiac pathological hypertrophy is associated with a significantly increased risk of coronary heart disease and has been observed in diabetic patients treated with rosiglitazone whereas most published studies do not suggest a similar increase in risk of cardiovascular events in pioglitazone-treated diabetic subjects. This study sought to understand the pathophysiological and molecular mechanisms underlying the disparate cardiovascular effects of rosiglitazone and pioglitazone and yield knowledge as to the causative nature of rosiglitazone-associated cardiac hypertrophy.

**Methods:**

We used a high-fat diet-induced pre-diabetic mouse model to allow bioinformatics analysis of the transcriptome of the heart of mice treated with rosiglitazone or pioglitazone.

**Results:**

Our data show that rosiglitazone and pioglitazone both markedly improved systemic markers for glucose homeostasis, fasting plasma glucose and insulin, and the urinary excretion of albumin. Only rosiglitazone, but not pioglitazone, tended to increase atherosclerosis and induced pathological cardiac hypertrophy, based on a significant increase in heart weight and increased expression of the validated markers, ANP and BNP. Functional enrichment analysis of the rosiglitazone-specific cardiac gene expression suggests that a shift in cardiac energy metabolism, in particular decreased fatty acid oxidation toward increased glucose utilization as indicated by down regulation of relevant PPARα and PGC1α target genes. This underlies the rosiglitazone-associated pathological hypertrophic cardiac phenotype in the current study.

**Conclusion:**

Application of a systems biology approach uncovered a shift in energy metabolism by rosiglitazone that may impact cardiac pathological hypertrophy.

## Background

Type 2 diabetes mellitus (T2DM) is a strong independent risk factor for premature death and disability from heart failure [1]. Managing T2DM and related heart failure is an ongoing concern and adequate treatment of T2DM remains an important issue. Thiazolidinediones (TZDs) are a class of drugs that initially showed great promise as oral therapy for T2DM. The TZDs, rosiglitazone and pioglitazone were widely used as hypoglycemic drugs in patients with T2DM, but due to a relatively high risk of adverse cardiac effects, specifically the use of rosiglitazone was banned in Europe in 2010 [2], whereas its use is restricted by the US Food and Drug Administration [3].

Despite their chemical and mechanistic similarities, there are large cohort studies suggesting that rosiglitazone causes heart failure to a greater extent than pioglitazone, yet provides no additional therapeutic benefit [2,4,5]. Questions as to how rosiglitazone may be leading to a relatively higher cardiovascular risk still remain largely unanswered. Insight into the regulatory mechanisms underlying the disparate cardiovascular effects of rosiglitazone and pioglitazone may have translational implications and yield knowledge as to the causative nature of TZD-associated cardiac events in man [6].

Rosiglitazone and pioglitazone are thought to exert their primary therapeutic effects through binding to the peroxisome proliferator-activated receptor gamma (PPARγ), thereby increasing sensitivity to insulin and producing better glycemic control [7]. Although PPARγ is most abundantly expressed in adipose tissue, it is widely expressed throughout the body [8], including the liver, skeletal muscle and, to a lesser extent, in the heart. There is increasing evidence that activation of PPARγ by TZDs could alter the transcription of hundreds of genes, only part of which contribute to glycemic control. It is conceivable that some of the other altered gene transcription cascades may lead to the development of cardiovascular complications. In addition, PPARγ-independent actions of TZDs have been suggested as a result of drug binding to cellular proteins different from PPARγ [4]. In this respect it might be significant that rosiglitazone-induced myocardial hypertrophy, an early hallmark and important risk factor for the development of heart failure [9], still occurs in cardiomyocyte-specific PPARγ knock-out mice [10].

In the present study we have used a systems biology approach for assessing similarities and differences between rosiglitazone and pioglitazone in gene expression profiles in the heart of a mouse model that mimics several of the characteristics of diabetes as observed in humans treated with rosiglitazone or pioglitazone. Bioinformatic analysis of expression profiles was used to identify regulatory pathways and upstream regulators that are influenced by the two TZD treatments. Similarities in expression patterns between the two drugs may reveal unknown mechanisms of action in addition to PPARγ binding, and possible sources of shared effects. Conversely, differences may reveal different regulatory events leading to the increased cardiovascular risk that may be unique to a specific glitazone. From the current study several findings emerged that provide insights into differential effects of rosiglitazone and pioglitazone within the cardiovascular system, including implications for compounding drug efficacy and cardiotoxicity.

## Methods

### Animals

The study was approved by the animal Ethics Committee of TNO, the Netherlands, and animal handling was performed according to the European directive on Laboratory Animals (86/609/EEC). Male LDLr deficient mice were bred in TNO facilities and housed in wire-topped Macrolon cages with a layer of sawdust as bedding, and diets and water were provided *ad libitum*.

### Study design and diets

The current study is part of a larger study, the design of which has been described in detail elsewhere [11]. In short, to induce characteristics of pre-diabetes, male LDLr deficient mice (n = 33) received Western type High Fat diet containing 24% (w/w) lard fat (Research diets D12541, USA), further referred to as High-Fat (HF), diet for 9 weeks. Then, mice were subdivided into three experimental groups and matched for plasma cholesterol and body weight (t = 0). One group (n = 15 mice, control) continued the HF-diet treatment, while the other two groups were fed an HF-diet containing rosiglitazone (0.01% w/w; Avandia, GSK) or pioglitazone (0.01% w/w; Actos, Takeda) for an additional 7 weeks. The daily doses of rosiglitazone and pioglitazone are 5.6 mg/kg body weight/day and 7.2 mg/kg body weight, respectively. In parallel to these HF-diet groups a separate control group was kept on chow for the duration of the experiment and served as a reference control for aging. Throughout the experiment all experimental diets were well tolerated and mice in the various treatment groups consumed comparable amounts of calories.

### Analysis of plasma glucose and insulin, and urinary albumin levels

Plasma was obtained via tail vein bleeding after 5 hours of fasting. Plasma glucose was quantified by the glucose hexokinase method (Instruchemie, Delfzijl, The Netherlands) and plasma insulin by ELISA (Ultrasensitive mouse insulin ELISA, Mercodia, Uppsala, Sweden). To assess glomerular barrier function, urinary albumin (Exocell Inc. Philadelphia, PA, USA) and creatinine concentrations were determined (Bethyl Laboratories Inc. Montgomery, TX, USA).

### Atherosclerotic lesion analysis

At the end of the study, mice were euthanized to collect hearts and aortas. Hearts were weighted and divided in two parts. The apex of the heart was snap-frozen in liquid nitrogen and stored at -80°C. The upper part was fixed in formalin and embedded into paraffin to prepare serial cross sections (5 μm-thick) throughout the entire aortic root area for histological analysis [12]. Cross-sections were stained with hematoxylin-phloxine-saffron (HPS) and atherosclerosis was analysed blindly in 4 cross-sections of each specimen (at intervals of 40 μm). An Olympus BX51 microscope and Cell^D software (Olympus, Zoeterwoude, The Netherlands) were used for morphometric computer-assisted analysis of lesion number and area.

### Nucleic acid extraction and microarray analysis

Nuclear acid extraction was performed as described previously in detail [13]. Briefly, total RNA was extracted from individual hearts (n = 5-6 hearts per experimental group, 16 samples in total, with normalized glucose levels at <13mM) using glass beads and RNAzol (Campro Scientific, Veenendaal, The Netherlands). RNA integrity was examined using the RNA 6000 Nano Lab-on-a-Chip kit and a bioanalyzer 2100 (Agilent Technologies, Amstelveen, The Netherlands). The Illumina^®^ TotalPrep™ RNA Amplification Kit (Ambion, art.No.AM-IL1791) was used to synthesize biotin labeled cRNA starting with 500 ng total RNA. The concentration of the labeled cRNA was measured using the Nanodrop spectrophotometer. The amount of biotinylated cRNA which was hybridized onto the MouseRef-8 Expression BeadChip was 750 ng. Illumina’s Genomestudio v1.1.1 software with the default settings advised by Illumina was used for Gene Expression analysis. All the quality control data of this BeadChip were within specifications of the microarray service provider (Service XS, Leiden, the Netherlands).

### Microarray data analysis

The probe-level background subtracted expression values were used as input for lumi package [14] of the R/Bioconductor (http://www.bioconductor.org; http://www.r-project.org) to perform quality control and a quantile normalization. Unexpressed probes (p > 0.01 in all experiments) were removed from further analysis, and 15725 probes remained in the analysis. Differentially expressed probes were identified using the limma package of R/Bioconductor [15]. The calculated P-values <0.01 were used as threshold for significance in cardiac tissue. Selected differentially expressed probes (DEPs) were used as an input for pathway analysis through Ingenuity Pathway Analysis suite (http://www.ingenuity.com, accessed 2013).

Upstream regulator analysis was performed using the Ingenuity Pathway Analysis (IPA) software. This analysis determines the activation state of transcription factors based on the observed differential gene expression. This results in an overlap p-value and activation z-score for each transcription factor in the IPA knowledgebase. The overlap p-value indicates the significance of the overlap between the known target genes of a transcription factor and the differentially expressed genes measured in an experiment. The activation z-score indicates activation (positive z-score) or inhibition (negative z-score) of a particular transcription factor. An activation z-score <-2 or >2 indicates significant activation or inhibition of a pathway or process.

### cDNA synthesis and quantitative real-time polymerase chain reaction

cDNA was synthesized from heart tissue RNA from mice in the HF + Rosi and HF + Pio groups with similar, i.e. normalized glucose levels. One microgram of total RNA was used for the high capacity RNA to cDNA kit (4387406, Applied Biosystems). Real time PCR was performed in triplicate on a Fast 7500 using the TaqMan gene expression assays (Applied Biosystems). Specific probes were applied to detect transcripts for B-type natriuretic peptide (*Bnp*;Mm01255770_g1 ) and A-type natriuretic peptide (*Anp*;Mm01255747_g1). The probes solute carrier family 27 (fatty acid transporter), member 1 (Slc27a1,Fatp; Mm00449511_M1), uncoupling protein 3 (Ucp3; Mm00494077_M1), Acyl-CoA dehydrogenase, very long chain (Acadvl Mm00444293_M1), and carnitine palmitoyltransferase 1b (Cpt1b; Mm00487200_M1) were applied to validate microarray results. All signals were compared to glyceraldehyde 3-phosphate dehydrogenase (*Gapdh*;4308313), hypoxanthine-guanine phosphoribosyltransferase (*Hprt*;Mm00446968_m1) and peptidylprolyl isomerase F (*Ppif*;Mm00506384_m1) as housekeeping genes.

### Statistics

All efficacy data are presented as mean ± standard deviation (SD). Data were analyzed using One-Way analysis of variance (ANOVA) and least significant difference (LSD) post hoc test unless stated otherwise. In all tests performed, the null hypothesis was rejected at the level of 5% probability (α = 0.05).

## Results

### Metabolic parameters

**Figure 1:**
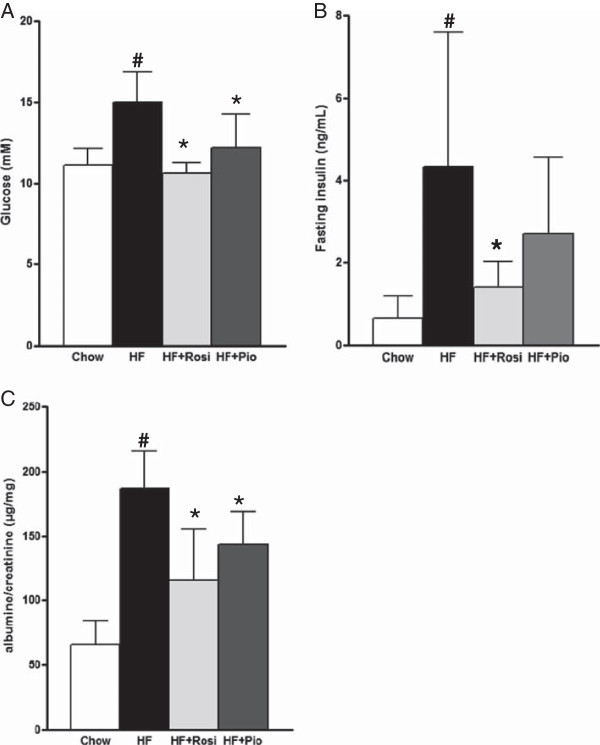
**Effect of drug treatment on metabolic parameters in LDLr-/- mice fed a chow diet or an HF-diet supplemented with rosiglitazone (HF + Rosi) or pioglitazone (HF + Pio).** Fasting plasma levels of glucose **(A)** and insulin **(B)**, and albumin/creatinin ratio in urine **(C)**. Values are mean ± SD. # indicates statistical significance P < 0.05 compared to chow, *indicates statistical significance P < 0.05 compared to HF-diet.

LDLr-/- mice fed an HF-diet over a 16-week period showed a significantly higher increase in body weight (15.8 ± 4.6 g) relative to mice on standard chow (3.2 ± 1.6 g; P < 0.001). The body weight gains of mice fed HF + Rosiglitazone (HF + Rosi) or HF + Pioglitazone (HF + Pio) tended to be even higher than those of HF fed mice (+14% and +10%, respectively; not statistically significant).HF-diet significantly increased plasma fasting glucose (15.0 ± 1.9 mM; P < 0.001; Figure 1A) compared to chow diet (11.3 ± 1.6 mM). HF + Rosi and HF + Pio treatment significantly reduced fasting glucose levels to 10.6 ± 0.7 mM (P < 0.01) and 12.2 ± 2.1 mM (P < 0.01), respectively. Fasting plasma insulin levels significantly increased with HF-diet to 4.3 ± 3.3 ng/ml vs. 0.7 ± 0.5 ng/ml with chow diet (P < 0.001; Figure 1B). HF + Rosi (1.4 ± 0.6 ng/ml; P < 0.01) and HF + Pio (2.7 ± 1.8 ng/ml; P = 0.09) treatment reduced fasting insulin levels. Urinary albumin/creatinine ratio, an indication for microalbuminuria, showed a 2.8-fold increase (186 ± 29 μg/mg; P < 0.05; Figure 1C) with HF-diet compared to chow diet (65 ± 19 μg/mg). Both HF + Rosi (116 ± 39 μg/mg; P < 0.05) and HF + Pio (143 ± 25 μg/mg; P < 0.05) significantly reduced the HF-diet-induced increase in albumin/creatinine ratio. In all, these data demonstrate that both HF + Rosi and HF + Pio improve metabolic parameters efficiently.

### Cardiac parameters

**Figure 2:**
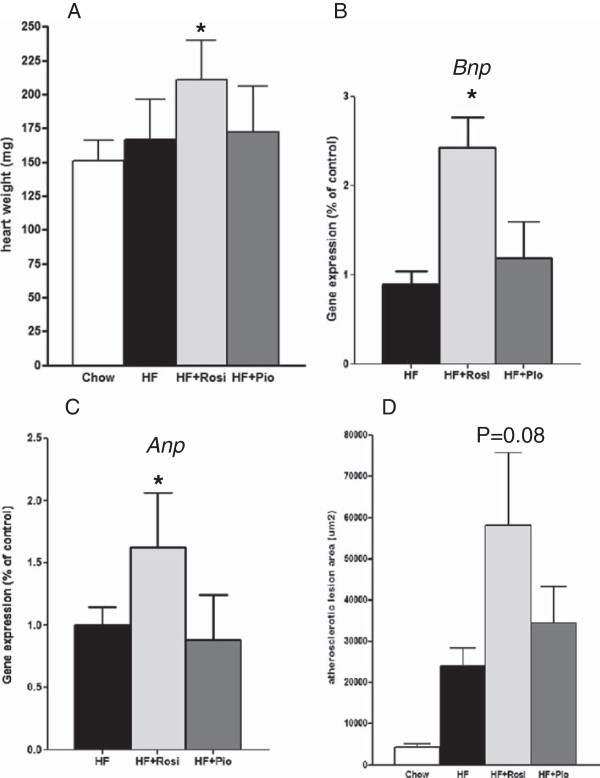
**Effect of drug treatment on cardiac parameters of LDLr-/- mice.** Heart weight **(A)**, gene expression of cardiac stress marker *Bnp***(B)**, and *Anp***(C)** assessed by RT-PCR, and quantitative analysis of atherosclerotic lesions in the aortic root area **(D)**. Values are mean ± SEM. # indicates statistical significance P < 0.05 compared to chow, *indicates statistical significance P < 0.05 compared to HF-diet.

HF-diet increased heart weight only slightly (167 ± 30 mg) compared to chow (151 ± 15 mg; not significant). Treatment with rosiglitazone significantly increased heart weight relative to HF (211 ± 29 mg; P < 0.05; Figure 2A). Also, the heart-to-body weight ratio (HW/BW) of HF + Rosi was increased compared with HF-diet (0.48% ± 0.06% vs. 0.39% ± 0.06%; P < 0.05). In contrast, pioglitazone did not affect heart weight (172 ± 34 mg) or HW/BW ratio (0.40% ± 0.06%) compared with HF. The increased heart weight in the HF + Rosi group was accompanied by an upregulation of the fetal/stress genes B-type natriuretic peptide (*Bnp*) (2.43 ± 0.33; P < 0.001; Figure 2B) and atrial natriuretic peptide (*Anp*) (1.62 ± 0.44; P < 0.01; Figure 2C), relative to the HF group (*Bnp,* 1.0 ± 0.14; *Anp,* 1.0 ± 0.14). Augmented expression of *Bnp* and *Anp* indicates pathological cardiac growth of the heart, which is an important and independent risk factor for cardiovascular diseases. Notably, *Bnp* (1.19 ± 0.41) and *Anp* (0.88 ± 0.36) transcript levels were not significantly altered with pioglitazone. Another common cause of cardiovascular disease is the development of atherosclerosis. Histological analysis of the aortic root showed that 16 weeks of HF-diet treatment alone mildly increased atherosclerosis development in LDLr-deficient mice (23,800 ± 4,460 μm^2^) compared with chow-fed mice (4,300 ± 850 μm^2^; P < 0.01). Rosiglitazone treatment tended to further increase total atherosclerosis area in the aortic root (58200 ± 17470 μm^2^; Figure 2D; P = 0.08), while HF + Pio showed no significant differences (34300 ± 8830 μm^2^). The question then arises what mechanistic processes could underlie these cardiac adverse effects of rosiglitazone. To address this question a genome-wide gene expression analysis of selected heart tissue samples was carried out.

### Genome-wide transcriptome analysis

**Figure 3:**
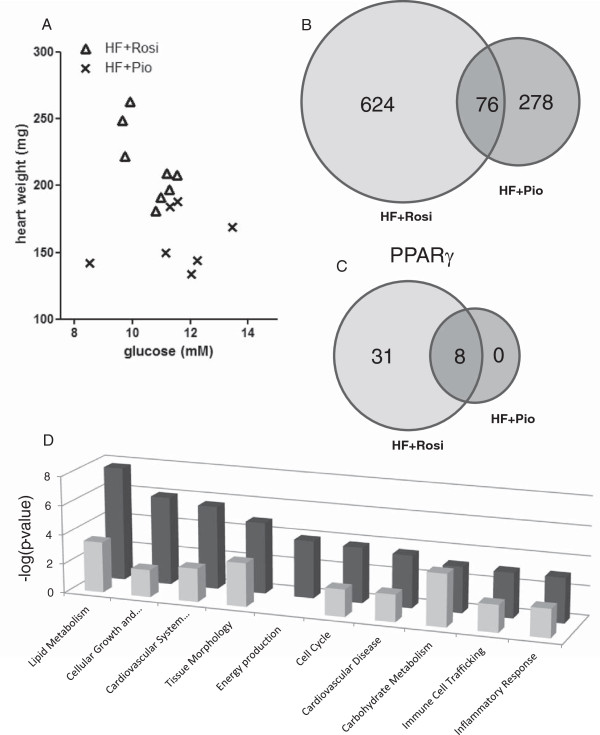
**Cardiac transcriptome analysis.** Relationship between fasting plasma glucose concentration and heart weight **(A)**, Venn-diagrams showing overlap in the total number of differentially expressed genes **(B)** and PPARγ target genes **(C)** in cardiac tissue in HF + Rosi and HF + Pio groups relative to HF-diet control group (P < 0.01). Graphical representation **(D)** of significantly enriched Biological Processes in HF + Rosi and HF + Pio treatment groups relative to HF-diet control group. Bars display a selection of significantly enriched biological processes (-log10 (p-value)) modulated by HF + Rosi (dark bars) or HF + Pio (light bars).

To elucidate which gene regulatory processes are modulated in the hearts of HF + Rosi and HF + Rio treated mice, transcriptome analysis of heart tissue was performed. Of note, heart tissue was used from mice that showed normalized plasma glucose levels (viz. below 13 mM) upon rosiglitazone or pioglitazone treatment (Figure 3A). Genes that were differentially expressed in the HF + Rosi (Additional file 1: Table S1) and HF + Pio (Additional file 2: Table S2) treatment groups relative to the HF-group were used for analysis of biological processes. The majority of the affected genes were modulated by rosiglitazone (700 genes compared to 345 genes with pioglitazone), and 76 genes were affected by both compounds (Venn-diagram; Figure 3B). Notably, only 39 genes of the genes affected by rosiglitazone are controlled by PPARγ and 8 of these were also affected by pioglitazone (Figure 3C). Together this shows that treatment with rosiglitazone, and to a lesser extent pioglitazone, is accompanied by substantial gene expression changes in the heart, but most of these changes are not direct related to PPARγ.

To get insight into potential changes of biological processes geneset enrichment analysis was performed. Sixty-seven (67) processes were significantly enriched by rosiglitazone and 49 processes by pioglitazone (Threshold p-value 0.001; Additional file 3: Table S3). The process “Carbohydrate metabolism” was affected by both drugs and to a comparable extent. All other processes including “Lipid Metabolism” and “Inflammatory Response” were affected by rosiglitazone to greater extent than by pioglitazone (Figure 3D). Of note, the process “Energy Production” was affected by rosiglitazone only.

**Figure 4:**
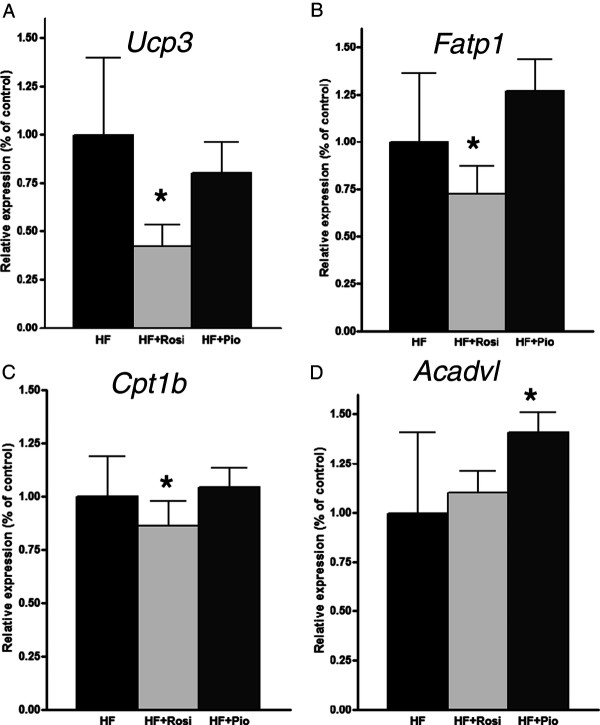
**Effect of drug treatment on gene expression changes.** Relative expression of genes Ucp3 **(A)**, Fatp1 **(B)**, Cpt1b **(C)**, and Acadvl **(D)** assessed by RT-PCR. Values are mean ± SD. *indicates statistical significance P < 0.05 compared to HF-diet.

**Figure 5:**
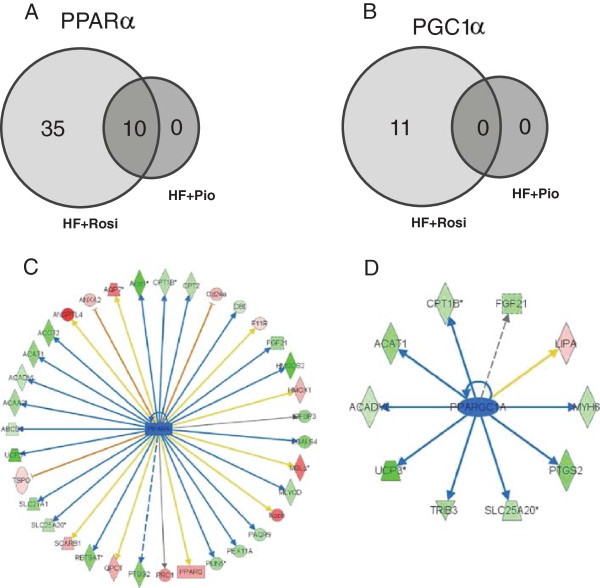
**PPARα and PGC1α target gene regulation.** Venn-diagrams displaying the number of target genes affected by nuclear transcription factor PPARα **(A)** and PGC1α **(B)**. PPARα **(C)** and PGC1α **(D)** target genes that are significantly upregulated (red) or downregulated (green) by rosiglitazone relative to HF-diet control (P < 0.01).

To further delineate the molecular processes that could underlie the adverse cardiac effects of rosiglitazone, we evaluated the rosiglitazone-specific genes of the Venn-diagram. *In silico* prediction of transcription factor activity based on the expression changes of known target genes revealed reduced activity of PPARα (p-value 3.4E-10; z-score -3.1) and PGC1α (p-value 1.1E-03; z-score -2.1) upon rosiglitazone treatment and an effect on target genes in the heart, including UCP3, FATP1, CPT-1, and ACADVL (Additional file 4: Table S4). The specific findings on the target genes from the transcriptome analysis are validated using RT-PCR method (Figure 4A-D). Functional geneset enrichment analysis of the dataset shows that these rosiglitazone-specific effects, involving PPARα and/or PGC1α-controlled genes, do at least partly explain the typical effects on the biological processes: Lipid Metabolism, Fatty Acid Metabolism and Energy Production (Venn diagrams; Figure 5A and B). Overall the effects of rosiglitazone on PPARα and PGC1α regulated genes indicate a strong down regulation of genes involved in Lipid Metabolism (Figure 5C) and Energy Production (Figure 5D) in the heart which might contribute to the observed pathological hypertrophy because such effects were not observed with pioglitazone.

## Discussion

The discrepancy in cardiovascular risk between patients taking rosiglitazone or pioglitazone and lack of insight into the underlying causes elicited the present study in mice [16,17]. Mouse heart tissue was used, since this allowed a comprehensive analysis of similarities and differences between rosiglitazone and pioglitazone. Bioinformatic analysis of the gene expression data identified regulatory pathways and upstream regulators that are specifically influenced by rosiglitazone.

In a high-fat diet-inducible model of combined hyperglycemia and cardiovascular disease we found that rosiglitazone and pioglitazone are effective glucose lowering drugs which markedly improved systemic markers of glucose homeostasis, fasting plasma glucose and insulin, and the urinary excretion of albumin. These findings are in line with observations made in humans [18,19] and other rodent models [20,21]. Importantly, only rosiglitazone treatment caused cardiac hypertrophy, defined as a significant increase in heart weight, and tended to increase atherosclerosis in the aortic root area. To our knowledge the disparate capacity of rosiglitazone and pioglitazone to induce cardiac hypertrophy was never studied before in a diet-induced mouse model. In general, two forms of cardiac hypertrophy can be distinguished, physiological and pathological hypertrophy. The latter is a significant independent risk factor for cardiovascular mortality and morbidity ([22], and references therein). Our study demonstrate that validated markers for pathological cardiac hypertrophy, ANP and BNP [23], were elevated by rosiglitazone but not by pioglitazone. This demonstrates that only rosiglitazone induced pathological cardiac hypertrophy in these mice within the study period of 7 weeks. Consistent with our observation in mice, rosiglitazone treatment also increased BNP plasma levels in T2DM patients without previous signs of cardiovascular disease [24], while pioglitazone did not have an effect on BNP levels [25].

Microarray technology is a powerful technique to analyze the effect of interventions on thousands of genes and across pathways [13]. Transcriptome analysis of the heart tissue revealed that, under conditions at which rosiglitazone and pioglitazone showed comparable hypoglycemic effects, 624 genes were significantly and differently altered by rosiglitazone. Only 31 of these are reportedly controlled by PPARγ. Furthermore, our analysis demonstrates that 354 genes are affected by pioglitazone, only 8 genes of which are target of PPARγ and all are in common with rosiglitazone. This implies, that in case the pathological cardiac hypertrophy is induced through a PPARγ-dependent mechanism, it could be caused by 31 rosiglitazone-modulated genes. However, the role of PPARγ in the development of pathological cardiac hypertrophy and in mediating the effect of rosiglitazone thereupon is not entirely clear yet. Several studies suggest that PPARγ is protective, as mice lacking PPARγ in the heart developed cardiac hypertrophy and dysfunction [26,27] and treatment with a PPARγ agonist reduced cardiac remodeling and fibrosis in a rat model of hypertension [27]. Yet, cardiac-specific over-expression of PPARγ in mice also resulted in cardiac dysfunction [28]. Another study demonstrated that transgenic mice with enhanced PPARγ activity developed concentric hypertrophy which progressed to dilated cardiomyopathy [29]. On the other hand, a study using cardiomyocyte-specific PPARγ knock-out mice indicated that rosiglitazone can promote the development of myocardial hypertrophy in a PPARγ -independent manner [10].

The heart relies on a constant high supply of energy, primarily met by the β-oxidation of fatty acids, to maintain the continuous contractile activity [30]. There is increasing evidence that a shift in cardiac energy metabolism towards decreased fatty acid oxidation and increased glucose utilization, can contribute to the development of pathological heart hypertrophy and failure [30]. Transcriptome analysis of the heart revealed that such a switch from fatty acid to glycolytic metabolism may also underlie the rosiglitazone-associated pathological hypertrophic cardiac phenotype in the current study. Rosiglitazone, but not pioglitazone, down regulated PPARα and PGC1α target genes. In PPARα-/- mice, cardiac hypertrophy is induced [31], and also in mice lacking PGC-1α, cardiac dysfunction becomes evident [32]. Functional enrichment analysis of the genes specifically affected by rosiglitazone indicates that the down regulated genes are related to biological processes ‘Lipid Metabolism’ and ‘Energy Production’ and included CPT1, VLCAD, and LCAD. Inhibition of CPT1 in cardiac tissue has been demonstrated to induce cardiac hypertrophy [33,34]. Similarly, cardiac hypertrophy was found in mice deficient in VLCAD or LCAD [35]. Further evidence for a putative role of PPARα in cardiac hypertrophy comes from studies on fibroblast growth factor 21 (FGF21), which is expressed in and released by cardiomyocytes through a PPARα - dependent mechanism [36]. Deficiency of FGF21 in the heart was demonstrated to induce of cardiac hypertrophy markers and reduce fatty acid oxidation [37].

## Conclusion

The mouse model of rosiglitazone-induced cardiac pathological hypertrophy combined with bioinformatics analysis of the transcriptome of the heart offers important insights into the pathophysiology of adverse cardiac hypertrophy. In particular, we uncovered a shift in cardiac energy metabolism from fatty acid oxidation toward increased glucose utilization and the roles of relevant PPARα and PGC1α target genes therein. These findings are also relevant for improving future drug efficacy void of cardiotoxicity.

## Competing interest

The authors declare that they have no competing interests.

## Authors’ contribution

LV, BvO, TeKo and RK conceived and designed the experiments. PYW, KS performed the experiments. LV, MR, ThKe analyzed the data. LV, BvO and TeKo wrote the manuscript. All authors read and approved the final manuscript.

## Pre-publication history

The pre-publication history for this paper can be accessed here:

http://www.biomedcentral.com/1755-8794/7/35/prepub

## Supplementary Material

Additional file 1: Table S1 Differentially expressed genes in HF+Rosi treated group relative to HF-group. Click here for file

Additional file 2: Table S2 Differentially expressed genes in HF+Pio treated group relative to HF-group. Click here for file

Additional file 3: Table S3 Biological processes significantly enriched in HF+ Rosi and HF+Pio relative to HF-group. Click here for file

Additional file 4: Table S4 Target molecules that are regulated by HF+Rosi and are predicted to be regulated by PPARα and/or PGC1α. Click here for file
